# Supplementary material for: Genetic Ablation of LAT1 Inhibits Growth of Liver Cancer Cells and Downregulates mTORC1 Signaling
Source: Int J Mol Sci. 2023 May 24;24(11):9171. doi: 10.3390/ijms24119171 (PMC10253063; doi:10.3390/ijms24119171)
Supplement: Supplementary file 1 [file ijms-24-09171-s001.zip › ijms-2396816-supplementary.pdf]

Supplement Figures

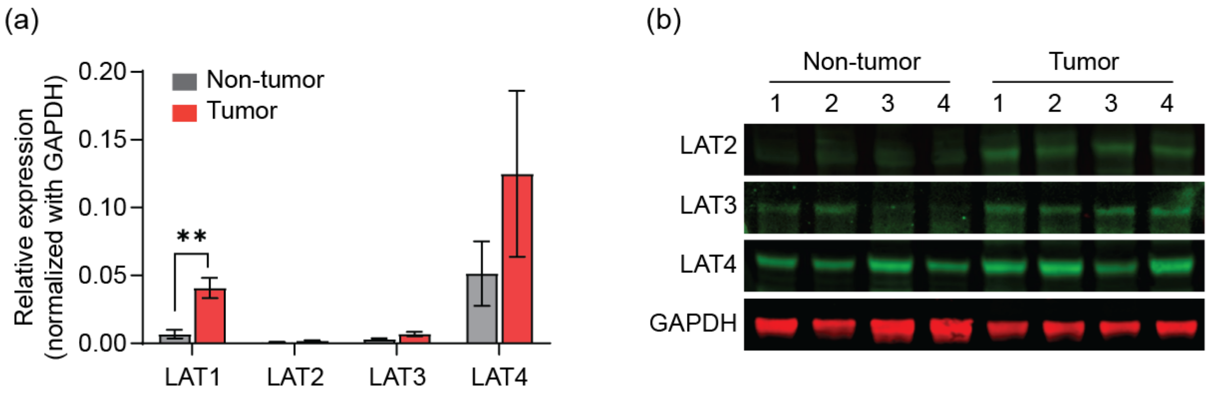

Figure S1. (a) qRT-PCR analysis of slc7a5, slc7a8, slc43a1, and slc43a2 genes in non-tumor liver and tumor liver of DEN mouse model. (b) Western blot analysis of LAT2, LAT3, and LAT4 in in non-tumor liver and tumor liver of DEN mouse model. **, $P < 0.01$

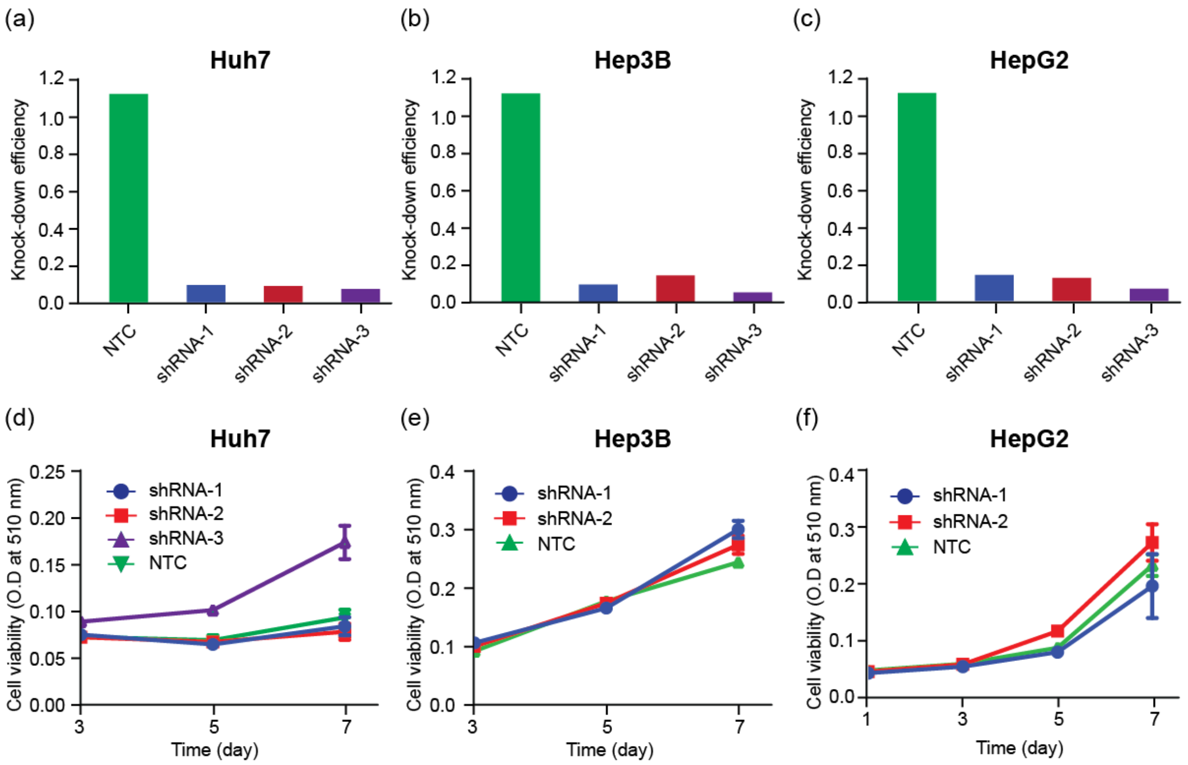

Figure S2. qRT-PCR results for LAT1 KD of different liver cancer call lines: (a) Huh7, (b) Hep3B, and (c) HepG2. SRB cell proliferation assays for LAT1 knock-down in different liver cancer cell lines: (d) Huh7, (e) Hep3B, and (f) HepG2. Even though there is relatively efficient KD for most shRNA constructs, there is no significant decrease in proliferation compared to non-targeting control.

(a)

Name	sgRNA sequence	Locaion
SLC7A5-1	CTTCACGCTGTAGCAGTTCA	Exon2
SLC7A5-2	GGCATCCTGGACCCGGGTGG	Exon2
SLC7A5-7	GAAGCCCAGCAGGATGATCA	Exon2

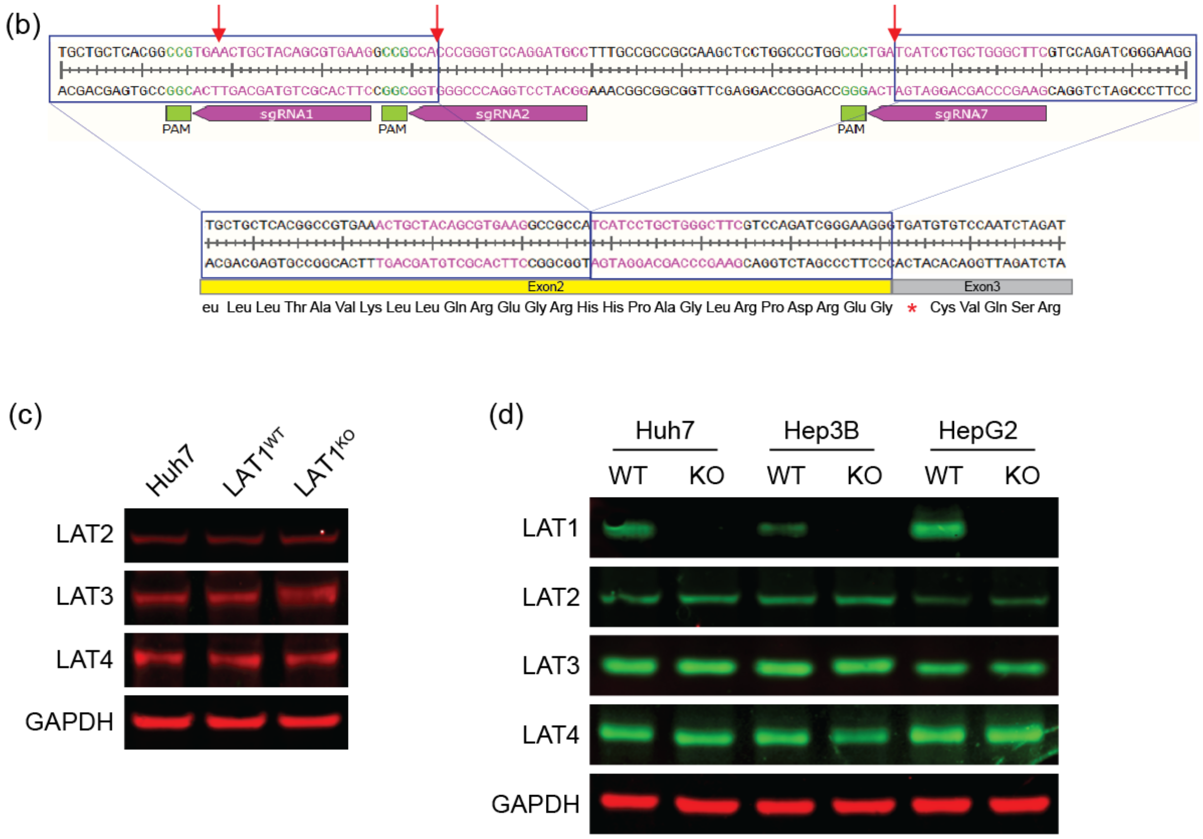

Figure S3. (a). sgRNA sequences of *slc7a5* gene. The sgRNA sequences were designed to target the second exon of the gene using CRISPR design tool and the resultant construct was subjected to Sanger sequencing to verify the correct sgRNA sequences. (b) Schematic representation of the mutation of *slc7a5*. HCC cell lines were each transfected with CRISPR/Cas9 vectors encoding three sgRNAs. To select LAT1 knock-out clones, candidate clones were examined by PCR analysis, sanger sequencing around the sgRNA targeted site. One successful clone has three cut sites and stop codon right after exon 2 of *slc7a5*. (c) Huh7, $LAT1^{WT}$, and $LAT1^{KO}$ were cultivated for 24 hours in normal DMEM and detected expression level of LAT2, LAT3, and LAT4 by western blot analysis. (d) LAT1 protein expression was completely knocked-out in $LAT1^{KO}$ of Huh7, Hep3B, and HepG2 cells, while LAT2, LAT3, and LAT4 proteins are expressed.

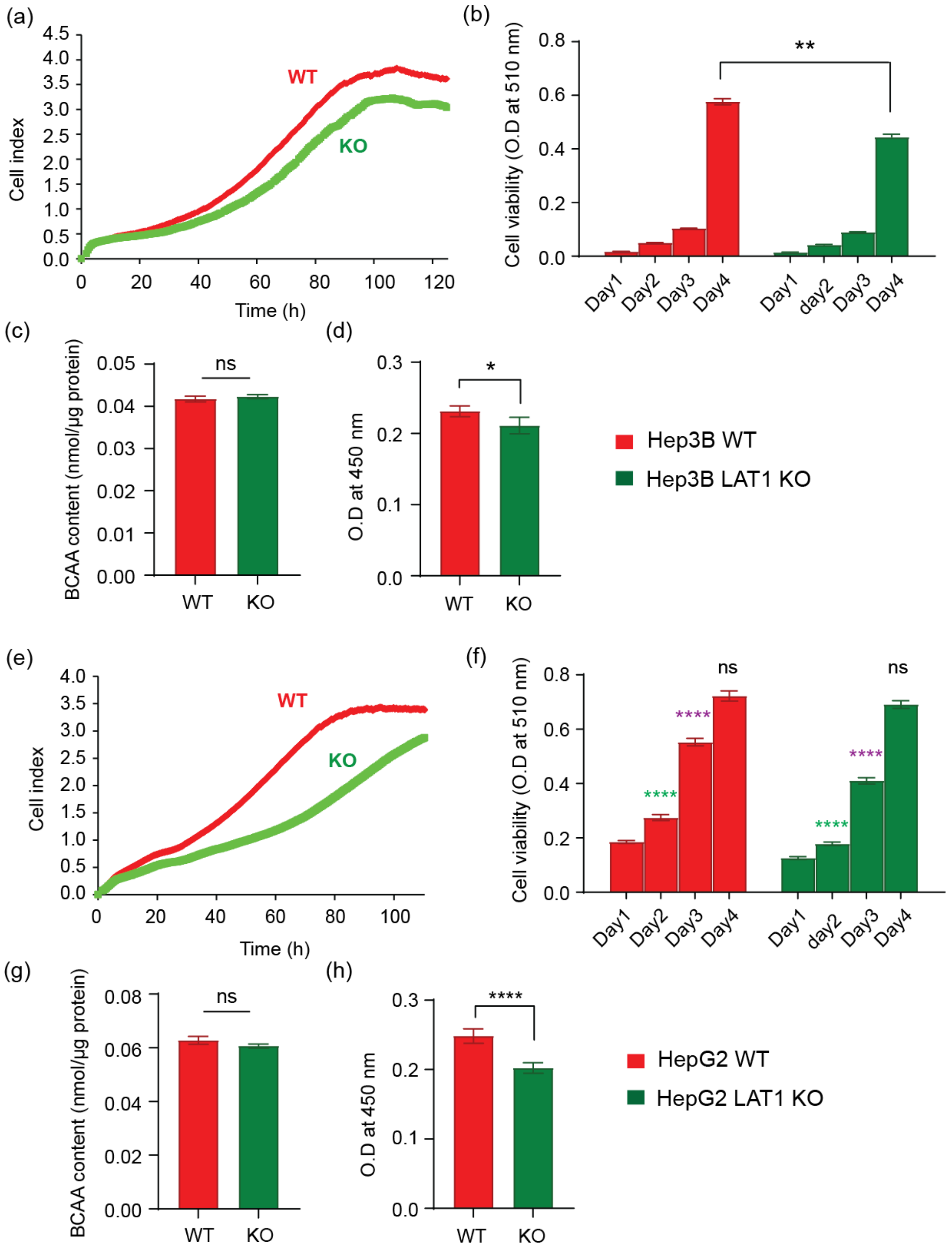

Figure S4. Real-time proliferation analysis (a, e), colorimetric SRB analysis (b, f), and BrdU incorporation assay (c, g) were performed to evaluate proliferation rates in the LAT1^{WT} and LAT1^{KO} of Hep3B (a, b, and c) and HepG2 (e, f, and g). (d, h) Intracellular BCAA content were measured in LAT1^{WT} and LAT1^{KO} of Hep3B (d) and HepG2 (h). The data represent are average of three independent experiments (n = 3) ± SEM. *, P < 0.05; **, P < 0.01; ****, P < 0.001

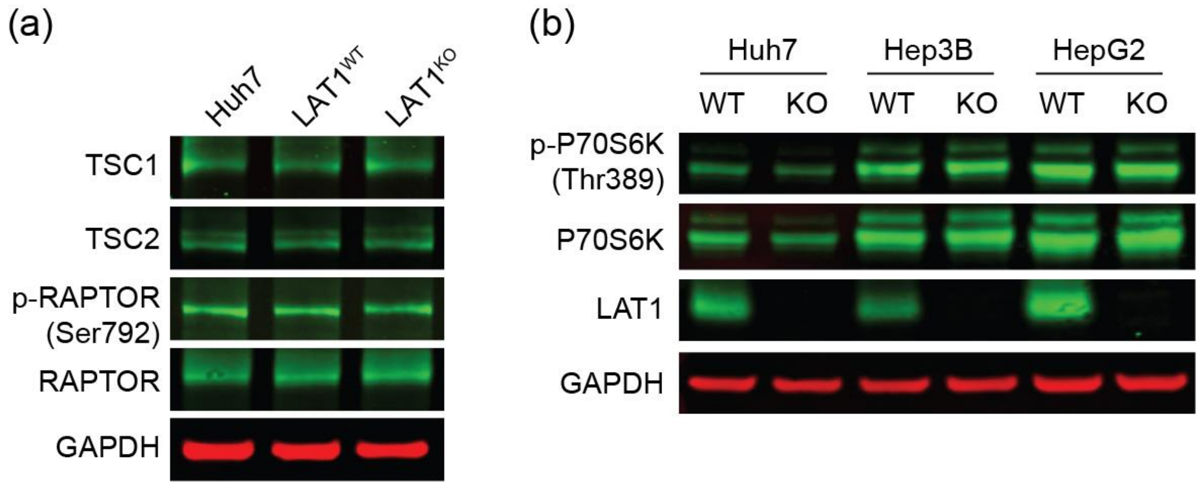

Figure S5. (a) Huh7, LAT1^{WT}, and LAT1^{KO} were cultivated for 24 hours in normal DMEM. Expression changes of TSC1, TSC2, RAPTOR, and p-RAPTOR were assessed by immunoblotting. (b) Phosphorylation and total p70S6K in the control LAT1^{WT} and LAT1^{KO} lysates of Huh7, Hep3B, and HepG2 cells were analysed by immunoblotting. GAPDH was used as a loading control.
